# Gold-catalyzed alkylation of silyl enol ethers with *ortho*-alkynylbenzoic acid esters

**DOI:** 10.3762/bjoc.7.76

**Published:** 2011-05-20

**Authors:** Haruo Aikawa, Tetsuro Kaneko, Naoki Asao, Yoshinori Yamamoto

**Affiliations:** 1 Department of Chemistry, Graduate School of Science, Tohoku University, Sendai 980-8578, Japan; 2 International Advanced Research and Education Organization, Tohoku University, Sendai 980-8578, Japan; 3 WPI-Advanced Institute for Materials Research, Tohoku University, Sendai 980-8578, Japan

**Keywords:** alkylation, gold catalysis, leaving group, silyl enol ether, substitution reaction

## Abstract

Unprecedented alkylation of silyl enol ethers has been developed by the use of *ortho*-alkynylbenzoic acid alkyl esters as alkylating agents in the presence of a gold catalyst. The reaction probably proceeds through the gold-induced in situ construction of leaving groups and subsequent nucleophilic attack on the silyl enol ethers. The generated leaving compound abstracts a proton to regenerate the silyl enol ether structure.

## Findings

**Scheme 1:**
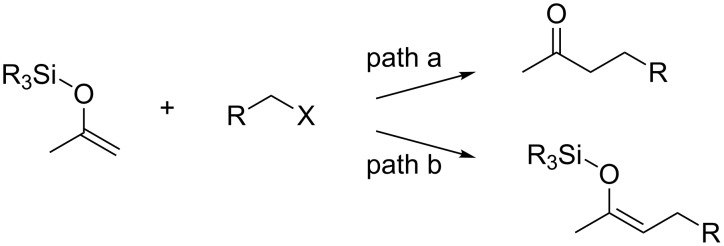
Alkylation of silyl enol ethers.

Silyl enol ethers have been widely used in organic synthesis as effective carbon nucleophiles for the construction of carbon frameworks [1–4]. Generally, they react with a variety of electrophiles to give carbonyl compounds as products due to cleavage of the silicon–oxygen bond. For example, the Lewis acid-catalyzed reaction of silyl enol ethers with alkyl halides is well known as one of the most efficient preparative methods for regio-defined α-alkylated ketones (path a in Scheme 1) [5–17]. In contrast, in this paper, we report a gold-catalyzed reaction of silyl enol ethers with *ortho*-alkynylbenzoic acid esters which leads to the formation of α-alkylated silyl enol ethers (path b).

We examined the reactions of silyl enol ether **1a** with *ortho*-alkynylbenzoic acid benzyl esters **2** in the presence of gold catalysts under several reaction conditions and the results are summarized in Table 1 [18–21]. With a cationic gold catalyst, derived from Ph_3_PAuCl and AgClO_4_, the reaction of **1a** with **2a** proceeded at 80 °C over 2 h and the benzylated silyl enol ether **3a** was obtained in 35% yield, along with the eliminated isocoumarin **4a** and recovered **2a** in 32% and 65% yields, respectively (entry 1). On the other hand, no products were obtained from the reaction of **1a** with benzyl benzoate (having no alkynyl group at the *ortho*-position) under similar reaction conditions. These results clearly show that the alkynyl moiety of ester **2a** is essential for the formation of **3a**. It is well known that concerted pericyclic ene-type reaction of silyl enol ethers with electrophiles, such as aldehydes or ketones, gives functionalized silyl enol ethers without desilylation [22–36]. To the best of our knowledge, however, this is the first example of the introduction of simple alkyl groups through a substitution-type reaction [37–40]. The chemical yield was increased to 55% by use of sterically hindered (*o*-Tol)_3_PAuCl as the gold catalyst (entry 2). Besides benzene, (CH_2_Cl)_2_ and 1,4-dioxane were also suitable solvents (entries 3 and 4). The use of 5 equiv of **1a** improved the chemical yield and **3a** was obtained in 72% yield (entry 5). The catalyst derived from AgOTf gave a better yield, although a longer reaction time was required (entry 6). Analogously, the reaction with **2b**, with a butyl group at the alkynyl terminus, gave **3a** in 75% yield (entry 7). In the current catalyst system using AgOTf, TfOH might be produced during the reactions due to the decomposition of AgOTf with a trace amount of water, which could be present in the reaction medium. However, the alkylation of **1a** with **2a** did not proceed at all with 5 mol % of TfOH. This result clearly indicates that the gold complex functions as a catalyst in the current transformations.

**Table 1:** Gold-catalyzed alkylation of silyl enol ether^a^.

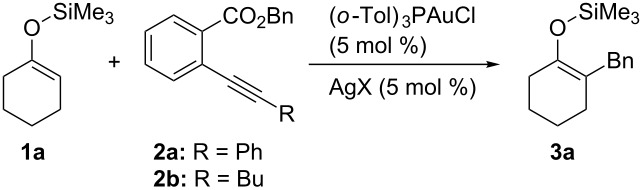					

Entry	**2**	AgX	Solvent	Conditions	Yield (%)^b^

1^c^	**2a**	AgClO_4_	benzene	80 °C, 2 h	35
2	**2a**	AgClO_4_	benzene	80 °C, 2 h	55
3	**2a**	AgClO_4_	(CH_2_Cl)_2_	80 °C, 2 h	44
4	**2a**	AgClO_4_	dioxane	100 °C, 2 h	58
5^d^	**2a**	AgClO_4_	dioxane	100 °C, 1 h	72
6^d^	**2a**	AgOTf	dioxane	100 °C, 10 h	80
7^d^	**2b**	AgOTf	dioxane	80 °C, 5 h	75

^a^Reaction conditions: 0.25 M solution of **2** was treated with **1a** (3 equiv) in the presence of the gold catalyst. ^b^NMR yield using CH_2_Br_2_ as an internal standard. ^c^Ph_3_PAuCl was used instead of (*o*-Tol)_3_PAuCl. ^d^5 equiv of **1a** was used.

We next examined the substrate generality with several types of silyl enol ethers **1** and esters **2** (Table 2). Treatment of five-membered silyl enol ether, cyclopentenyloxytrimethylsilane (**1b**), with **2b** in the presence of the gold catalyst gave the corresponding benzylated product **3b** in 61% yield (entry 1). It is worth mentioning that benzo-fused silyl enol ether **1c** is suitable for this transformation as shown in entries 2 and 3, whereas it cannot be used for ene-reaction due to the lack of a hydrogen atom at the α’-position. Not only cyclic silyl enol ethers but also an acyclic silyl enol ether underwent the reaction. Thus, **1d** reacted stereoselectively with **2a** to yield *E*-**3e**. Interestingly, the formation of the isomeric *Z*-**3e** was not detected at all (entry 4) [41]. The reaction of **1a** with allyl ester **2d** proceeded smoothly and the corresponding allylated product **3f** was obtained in 70% yield (entry 5) [42].

**Table 2:** Gold-catalyzed alkylation of silyl enol ether^a^.

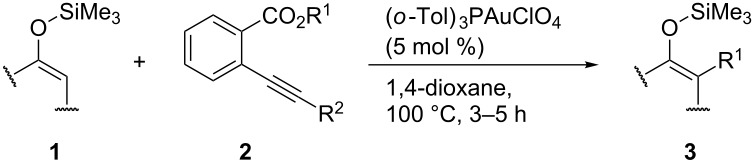							

Entry	**1**		**2**	R^1^	R^2^	**3**	Yield (%)^b^

1^c^	**1b**	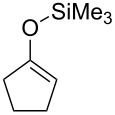	**2b**	Bn	Bu	**3b**	61
2	**1c**	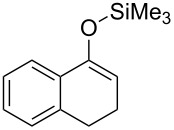	**2b**	Bn	Bu	**3c**	70
3^d^	**1c**	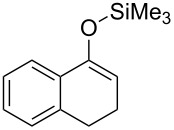	**2c**	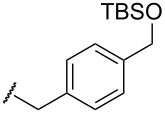	Ph	**3d**	60^e^
4^c,f^	**1d**	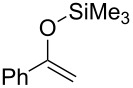	**2a**	Bn	Ph	**3e**	61
5	**1a**	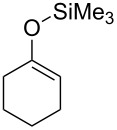	**2d**	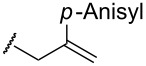	Ph	**3f**	70

^a^Reaction conditions: 0.25 M solution of **2** was treated with **1** (5 equiv) in the presence of the gold catalyst. ^b^NMR yield using CH_2_Br_2_ as an internal standard. ^c^10 mol % of the catalyst was used. ^d^3 equiv of **1** was used. ^e^Yield of isolated product. ^f^AgOTf was used instead of AgClO_4_.

**Scheme 2:**
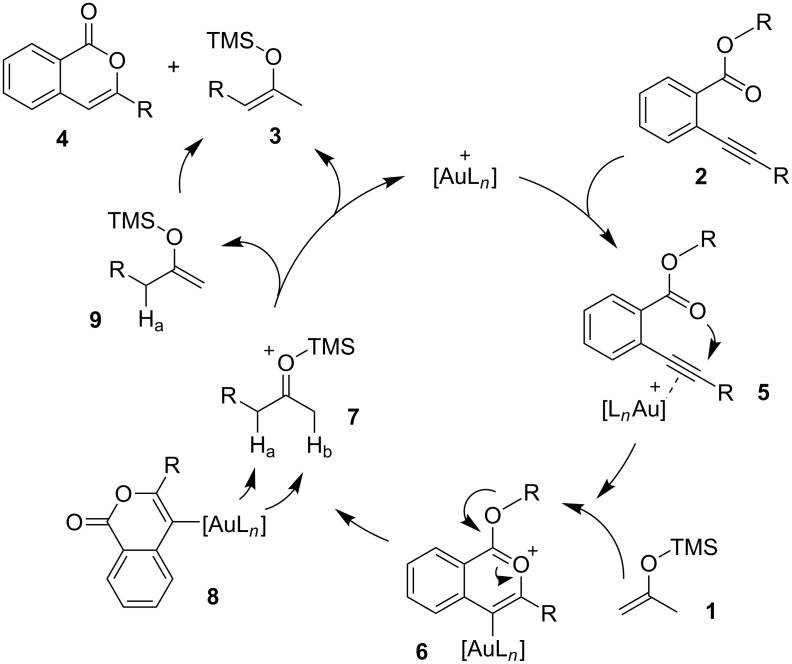
Plausible mechanism for the alkylation of silyl enol ether.

A plausible mechanism for the gold-catalyzed alkylation of silyl enol ethers is shown in Scheme 2. The gold catalyst enhances the electrophilicity of the alkynyl moiety of **2**, leading to the formation of a cationic intermediate **6** via the intramolecular nucleophilic attack of the carbonyl oxygen on the alkyne as shown in **5**. Due to the high leaving ability of the isocoumarin moiety of **6**, the silyl enol ether **1** attacks the R group to give the intermediate **7** together with the gold complex **8** as a leaving compound [43–46]. In the case of ordinary substitution reactions with alkyl halides (path a in Scheme 1), generated halide ions would attack the silyl group, due to their strong affinities with the silicon atom, and cleave the silicon–oxygen bond of **7**. However, in the present reaction system, intermediate **8** would prefer to act as a base and abstract a proton, H_a_, from the α-position rather than attack the silyl group as a nucleophile, probably due to steric and electronic reasons. For these reasons, deprotonation of **7** occurs to give the product **3** together with **4** as a final leaving compound.

**Scheme 3:**
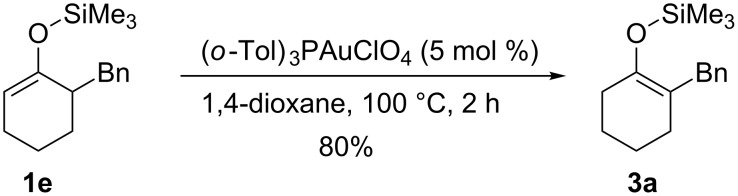
Gold-catalyzed isomerism of silyl enol ether.

**Scheme 4:**
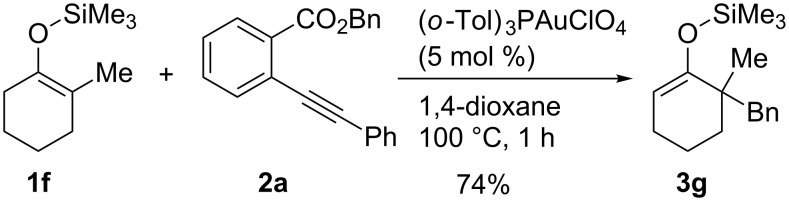
Gold-catalyzed alkylation of tetra-substituted silyl enol ether.

On the other hand, in the case of reactions with silyl enol ethers having a proton, H_b_, at the α’-position, compound **9** might be produced through the deprotonation of H_b_ by **8**. However, such products were not obtained in any of the examples studied. These results imply that isomerism from **9** to **3** would occur during the reaction. Thus, compound **1e** was prepared according to a known procedure and treated with the gold catalyst at 100 °C for 2 h (Scheme 3). As expected, the isomerization of the double bond occurred and **3a** was obtained in 80% yield. This result shows that the indirect pathway from **7** to **3** via deprotonation of H_b_ is also possible. In addition, it was found that the reaction of **1f**, having no hydrogen at the α-position, proceeded smoothly and α,α-dialkyl silyl enol ether **3g** was obtained in good yield (Scheme 4). Obviously, this result supports the possibility of the indirect pathway.

In conclusion, we have developed an unprecedented alkylation method for silyl enol ethers, using a gold catalyst and *ortho*-alkynylbenzoic acid esters as alkylating agents. The reaction probably proceeds through the gold-induced in situ construction of a leaving group and subsequent nucleophilic attack on the silyl enol ether. Unlike ordinary leaving groups, such as halide ions, the generated leaving compound **8** acts as a base and abstracts a proton to regenerate the silyl enol ether structure. The current protocol can also be used with substrates having no hydrogen at the α-position, such as **1f**. Further studies to elucidate the mechanism of this reaction and to extend the scope of synthetic utility are underway.
